# Auditory Localisation Biases Increase with Sensory Uncertainty

**DOI:** 10.1038/srep40567

**Published:** 2017-01-11

**Authors:** Sara E. Garcia, Pete R. Jones, Gary S. Rubin, Marko Nardini

**Affiliations:** 1 University College London, UCL Institute of Ophthalmology, London, UK; 2 NIHR Moorfields Biomedical Research Centre, London, UK; 3 Durham University, Department of Psychology, Durham, UK

## Abstract

Psychophysical studies have frequently found that adults with normal hearing exhibit systematic errors (biases) in their auditory localisation judgments. Here we tested (i) whether systematic localisation errors could reflect reliance on prior knowledge, as has been proposed for other systematic perceptual biases, and (ii) whether auditory localisation biases can be reduced following training with accurate visual feedback. Twenty-four normal hearing participants were asked to localise the position of a noise burst along the azimuth before, during, and after training with visual feedback. Consistent with reliance on prior knowledge to reduce sensory uncertainty, we found that auditory localisation biases increased when auditory localisation uncertainty increased. Specifically, participants mis-localised auditory stimuli as being more eccentric than they were, and did so more when auditory uncertainty was greater. However, biases also increased with eccentricity, despite no corresponding increase in uncertainty, which is not readily explained by use of a simple prior favouring peripheral locations. Localisation biases decreased (improved) following training with visual feedback, but the reliability of the visual feedback stimulus did not change the effects of training. We suggest that further research is needed to identify alternative mechanisms, besides use of prior knowledge, that could account for increased perceptual biases under sensory uncertainty.

In daily life we are constantly surrounded by sounds originating from different locations within our environment, and we are usually able to estimate the source of the sound without any noticeable bias. However, psychophysical studies have found that, when asked to align a visual stimulus with a sound source, human adults show systematic errors in their judgments. Many studies report overestimations of the azimuth of a sound source^1^,^2^, though underestimations have been documented too^3^. Here we tested whether (1) such biases might indicate reliance on a “prior” for sound location, in line with Bayesian Decision Theory^4^ (detailed below), and whether (2) subjects can learn to reduce such biases after training with accurate visual feedback.

Lewald and Ehrenstein^1^ asked healthy participants (with normal sight and hearing) to adjust the position of a laser spot toward the perceived location of a band-pass filtered noise. Results indicated a general tendency to overestimate auditory eccentricity, with greater overestimations for more eccentric sound sources (reaching up to 10.4 degrees overestimation at 22 degrees eccentricity). In addition to eccentricity^1^,^5^, auditory localisation errors have been found to vary in magnitude according to the elevation^6^, frequency^1^, or bandwidth^7^ of the auditory stimulus, and according to the participant’s age^2^, eye^8^,^9^ or head^10^ position, and method of response^10^.

Differences in patterns of systematic error across response methods, or different eye and head positions, suggest that biases in somatosensory or visual modalities may contribute to the auditory localisation bias. For example, participants asked to point toward a transient visual stimulus have been found to overestimate the target’s position when fixating straight ahead, but to underestimate it when eye movements toward the target are allowed^11^ (but note biases independent of eye movements have also been reported^12^). However, regardless of the direction, size, or cause of the visual-auditory misalignments measured, the question arises as to why any perceived systematic discrepancy exists? Visual and auditory space have different frames of reference: visual space is initially eye-centred, based on direct projections to the retina, whereas auditory space is initially head-centred, computed from binaural differences and spectral cues. Consequently, one proposed explanation is that auditory-visual spatial mismatches arise due to shortcomings in accounting for the position of the eyes relative to the head when relating auditory to visual representations of location^13^,^14^. However, systematic errors have been documented even when the eyes remain stationary at straight ahead with respect to the head^1^,^3^,^14^. Moreover, humans continuously receive visual and auditory feedback from their environment, which should enable them to detect and correct for misalignments in cross-modal spatial representations. For example, wearing prism glasses causes the visual field to shift, altering the visual-motor mapping, but after a brief period of exposure, human adults quickly adapt to the visual displacement^15^. Hence, it is unclear why humans would not learn to similarly adapt or ‘recalibrate’ their visual-auditory mapping, so as to reduce any spatial inconsistencies.

Human perception and action are increasingly understood in terms of Bayesian Decision Theory (BDT^16^,^17^,^18^;). There is evidence that the nervous system reduces the overall uncertainty of estimates by combining multiple sensory estimates both with each other and also with prior knowledge^18^,^19^,^20^. The ideal (Bayesian) observer would change their relative reliance on each estimate (e.g. on vision vs. audition^19^), and on prior knowledge, as the relative reliabilities (i.e. signal-to-noise ratios) of each changes. This theoretical prediction is frequently met by behavioural data^18^,^19^,^20^, and also, more recently, by neuronal representations in animal models^21^.

The BDT framework has provided novel explanations of several important perceptual phenomena. For example, the classic effect of “visual capture” on non-visual perception in multisensory tasks has been shown to reverse when the relative cue reliabilities change^19^,^20^; and this behaviour is also observed in a BDT idea-observer, for whom perception is driven by the reliability-weighted average of each sensory estimate. Additionally – and of direct relevance to the present study – previously unexplained *systematic biases* in perception have been proposed to reflect Bayesian reliance on prior knowledge. For example, humans’ systematic underestimation of the speed of moving objects has been explained by use of a “slow motion prior”, reflecting the real-world statistics that objects are most likely to be static in natural visual scenes^22^,^23^. More recently, an equivalent slow-motion prior, leading to underestimations in the speed of sound motion has been reported^24^.

Evidence that systematic biases reflect the use of Bayesian priors come from findings that biases increase as sensory uncertainty increases. For example, the speed of a lower-contrast visual motion stimulus is more difficult to judge precisely and is also perceived as slower^25^, consistent with increased reliance on a prior for slow speeds^22^. In the present study we asked whether a similar approach might account for long-documented but, as yet, unexplained biases in human auditory localisation. Specifically, we tested, to our knowledge for the first time, whether auditory localisation biases increase as sensory uncertainty increases (i.e. signal-to-noise ratio decreases). Such a result would be consistent with use of a Bayesian prior for the eccentricity of sound sources.

A second aim of our study was to ask whether auditory localisation biases could be reduced after training with accurate visual feedback, and if so, whether feedback reliability impacted learning. Previous studies have found that sound localisation biases introduced experimentally by manipulating auditory cues are reduced following training^26^,^27^,^28^. To account for a bias, the perceptual system needs to identify the cause of the error as systematic as opposed to random (reflecting uncertainty in the sensory representation or prior). In line with this, adults have been found to more quickly reduce systematic errors in their motor reaching responses when the position of visual feedback was more certain^29^. Here we tested whether the effect of training on reductions in auditory localisation errors would similarly depend on the reliability of visual feedback. If participants were using visual feedback to adjust systematic errors (in the prior or sensory representation) we would expect participants trained with more reliable visual feedback to show greater improvements in accuracy. Alternatively, improvements in auditory localisation could reflect reduced reliance on prior knowledge due to changes in the uncertainty of the prior or sensory representation.

## Results

First we considered whether biases in auditory localisation might be explained by the existence of a Bayesian prior. According to BDT, the influence of prior knowledge should increase when sensory information is less reliable. To test this, we measured participants’ auditory and visual localisation biases and variability, before any training, and assessed whether biases in auditory localisation increased as the signal-to-noise ratio (reliability) of the auditory stimulus decreased.

### Variability and bias before training

**Figure 1:**
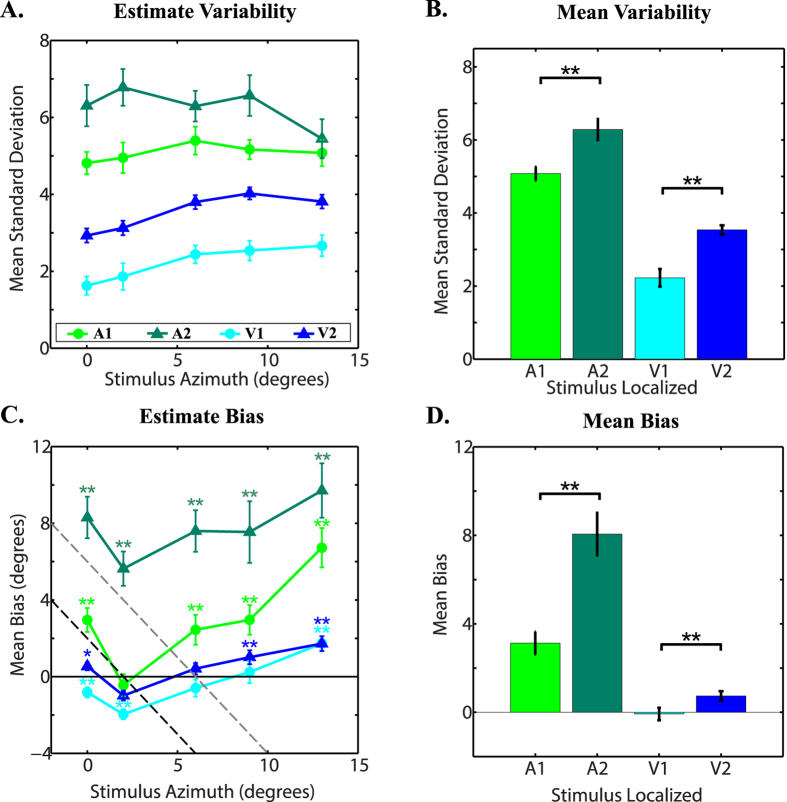
Bias and Variability in Localisation of Auditory and Visual Stimuli Before-Training. (**A**) Mean variability at each location for each stimulus. (**B**) Mean variability across locations for each stimulus. *95% CI excludes 0; **99% CI excludes 0. (**C**) Mean bias at each location for each stimulus. **Means differ significantly on paired t-test with p < 0.01. (**D**) Mean bias for each stimulus across all locations tested. Grey dotted line in C indicates the line predicted by responding according to the mean of the target stimulus set. Black dotted line in C indicates the line predicted by responding according to the mean of the speakers presenting background noise. Error bars represent the standard error of the mean.

Participants localised “more reliable” (A1, V1) and “less reliable” (A2, V2) auditory and visual stimuli. To manipulate the reliability of visual and auditory localisation, the background noise level was increased and the number of visible LEDs was reduced (see Method for details). Figure 1 shows mean auditory and visual localisation variability for each of the locations at which stimuli were presented (A) and across all locations (B). As intended, increasing the background noise level significantly increased the variability (i.e. reduced the reliability) of auditory localisation (σ_A2_ > σ_A1_, *t*_[23]_ = 5.88, *p* < 0.001). Similarly, reducing the number of visible LEDs significantly increased the variability of visual localisation (σ_V2_ > σ_V1_, *t*_[23]_ = 7.94, *p* < 0.001). Hence, our cue manipulations succeeded in varying sensory uncertainty.

Figure 1C shows mean auditory and visual localisation biases at each of the locations at which stimuli were presented. Participants tended to overestimate the eccentricity of auditory stimuli at each of the locations tested, especially the “less reliable” auditory stimulus A2. Estimates of visual stimuli were more accurate. Biases are not explained by participants’ responding towards the mean location of the target stimulus set (depicted in Fig. 1C by the grey dashed line) or the mean location of the background speaker set (depicted in Fig. 1C by the black dashed line).

If participants combined sensory evidence with prior knowledge in the manner of an ideal (Bayesian) observer, they would weight prior knowledge more as sensory evidence becomes less reliable (more uncertain). Hence, if biases in localisation reflect (at least partly) the use of a prior, biases would be expected to increase as the reliability of sensory information decreases. As Fig. 1D shows, auditory localisation biases were significantly greater for the less reliable auditory cue than more reliable auditory cue (μ_A2_ > μ_A1_, *t*_[23]_ = 7.56, *p* < 0.001). Mean biases (across locations) were also significantly greater for the less reliable visual cue than more reliable visual cue (μ_V2_ > μ_V1_, *t*_[23]_ = 2.83, *p* = 0.009), however, participants did not consistently overestimate the azimuth of less/more reliable visual stimuli across all locations tested (see Fig. 1C).

### Effects of eccentricity on variability and bias

In addition to the overall effects described in Fig. 1B and 1D, we also looked at the patterns of variability and bias across different locations (azimuths); Fig. 1A and 1C. Repeated-measures ANOVAs with stimulus reliability (A1, A2 or V1, V2) and azimuth (0, 2, 6, 9, 13 degrees) as within-subject factors were used to assess whether there was any effect of azimuth, or any interaction between reliability and azimuth, on localisation variability or bias. Where the assumption of sphericity was not met, Greenhouse-Geisser corrected values are reported.

For *localisation variability* with the auditory stimuli (A1, A2; Fig. 1A), a repeated-measures ANOVA indicated that there was a significant effect of stimulus reliability (*F*_[1, 22]_ = 31.09, *p* < 0.001), but no significant effect of azimuth (*F*_[2.78, 61.24]_ = 1.02, *p* = 0.385) and no significant interaction between reliability and azimuth (*F*_[2.61, 57.43]_ = 1.52, *p* = 0.224). For localisation variability with the visual stimuli (V1, V2; Fig. 1A), a repeated-measures ANOVA indicated that there was a significant effect of both stimulus reliability (*F*_[1, 23]_ = 63.00, *p* < 0.001) and azimuth (*F*_[4, 92]_ = 17.27, *p* < 0.001), but no significant interaction between reliability and azimuth (*F*_[4, 92]_ = 0.53, *p* = 0.715). Besides the main effects of our reliability manipulations (also seen in Fig. 1B), this shows (i) for audition, no further effect or interaction related to azimuth, i.e. similar variability across azimuths, whereas (ii) for vision, a main effect related to azimuth, with reliability decreasing away from central vision, as might be expected.

For *localisation bias* with the auditory stimuli (A1, A2; Fig. 1C), a repeated-measures ANOVA showed a significant effect of stimulus reliability (*F*_[1, 22]_ = 54.24, *p* < 0.001) and azimuth (*F*_[1.79, 39.28]_ = 5.08, *p* = 0.013), but no significant interaction between stimulus and azimuth (*F*_[2.70, 59.32]_ = 2.55, *p* = 0.070). For localisation bias with the visual stimuli (A1, A2; Fig. 1C), there was a significant effect of stimulus reliability (*F*_[1, 23]_ = 8.03, *p* = 0.009) and azimuth (*F*_[1.89, 43.35]_ = 14.05, *p* < 0.001), but no significant interaction between reliability and azimuth (*F*_[2.63, 60.37]_ = 2.33, *p* = 0.091). Besides the main effects of our reliability manipulations (also seen in Fig. 1D), this shows that for both audition and vision the degree of bias changed with eccentricity (main effect of azimuth), but in a similar manner for both reliability levels (no interaction).

### Accounting for patterns of bias

With the exception of the 0 degrees location – which may be atypical, as it corresponds with the location of the fixation point – biases tended to increase with azimuth (Fig. 1C). According to Bayesian principles, this could be explained by a non-uniform prior (i.e., one that favoured more eccentric responses), combined with a situation in which sensory uncertainty increased as one progressed further from the azimuth (i.e., resulting in increasing reliance on the prior). However, in the case of auditory stimuli, uncertainty (variability) was quite stable (Fig. 1A), yet bias increased with azimuth (Fig. 1C). Specifically, post hoc comparisons with a Bonferonni adjustment showed that auditory localisation bias was significantly greater for stimuli presented at 13 degrees, than stimuli at 2 (*p* = 0.034), 6 (*p* = 0.022) and 9 (*p* = 0.040) degrees. Another explanation for non-uniform biases is that bias should be lower at locations favoured by the prior. However, the pattern of auditory biases in Fig. 1C also seems to run counter to this possibility: biases indicate overestimations in eccentricity (suggesting a prior shifted away from the centre), yet biases were greatest at the highest eccentricity – and so did not decrease, but *increased*, away from the centre.

A simple weighted linear combination of an unbiased Gaussian sensory likelihood and prior would predict that bias *b* varies with azimuth *A* by


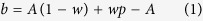


where *p* is the azimuth of the prior and *w* is the weight given to the prior – this simplifies to


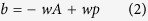


It is clear that this linear equation cannot produce positive gradients of the kind seen in Fig. 1C, since the weight term *w* has to be positive. The gradient is set by –*wA*, so when *w* is positive, the gradient *–wA* is negative.

We considered whether more complex interactions with, or forms of, the prior might explain these data, but were unable to formulate any that were convincing. For example, in “robust cue integration”^30^, estimates (here, the likelihood and the prior) are combined less as the conflict between them increases. However, for an effect of this kind to reverse the expected gradient so that priors increase (rather than decrease) towards the location of the prior (Fig. 1C) there would have to be extremely drastic changes in weighting for the prior over a very short span of azimuths. Our conclusion, therefore, is that although localisation biases increased with uncertainty, use of a prior in the BDT framework does not readily explain the entire pattern of results. For auditory localisation, bias appears to be a function both of the strength of the likelihood, and the absolute spatial location of the estimate.

### Effects of training with feedback

To test whether sound localisation biases can be reduced with experience, we compared sound localisation during and after completion of auditory localisation with visual feedback, and compared biases and variability to before-training results.

Before-, during-, and after- training, all participants were asked to localise the less reliable auditory stimulus (A2); see Table 1. During training, half the participants received more reliable visual feedback (VF1), and half received less reliable feedback (VF2). The reliability of the visual feedback was manipulated to assess whether this affected the degree of any improvement in sound localisation accuracy. Errors in localisation could be systematic (bias), random (sensory uncertainty), or both. An ideal system would adapt more quickly to feedback that is more likely to reflect systematic than random errors^29^. If visual feedback were used to adjust inaccuracies, we might expect a more reliable visual feedback cue to lead to a greater reduction in bias. Repeated-measures ANOVAs with experimental phase (before-, during-, after- training) as the within-subjects factor and visual feedback reliability (VF1, VF2) as the between-subjects factor were run to assess whether phase or visual feedback reliability had an impact on A2-localisation bias or variability.

### Bias with the trained auditory stimulus

**Figure 2:**
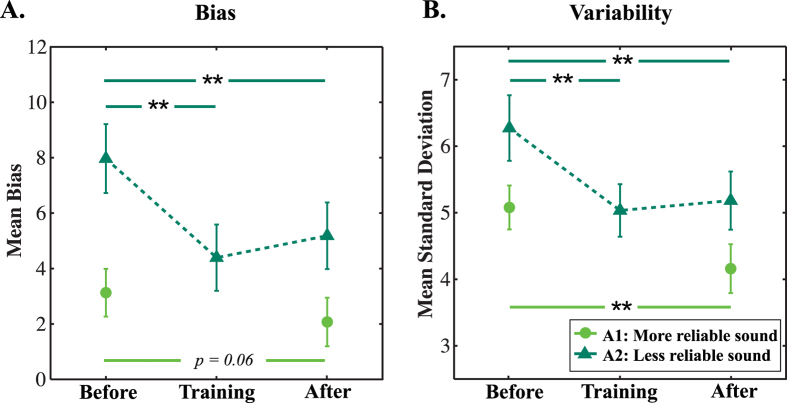
Mean Bias (**A**) and Mean Variability (**B**) for the Different Experimental Phases and Auditory Stimuli. Bars represent standard error of the mean. Paired-sample t-test results: *p < 0.05; **p < 0.01.

As bias data did not meet the assumption of sphericity (χ^2^_[2]_ = 12.72, p = 0.002), Greenhouse-Geisser corrections are reported. Experimental phase had a significant effect on bias (*F*_[1.38,30.26]_ = 12.44, *p* < 0.001), while neither feedback reliability (*F*_[1,22]_ = 0.02, *p* = 0.896) nor the interaction (*F*_[1.38,30.26]_ = 0.97, *p* = 0.360) did. Figure 2A shows the mean bias for each of the experimental phases. Post hoc comparisons with a Bonferonni adjustment showed that bias was significantly reduced in training (*t*_[23] =_ 4.02, *Bonferonni-corrected p* = 0.002) and after-training (*t*_[23] =_ 3.26, *Bonferonni-corrected p* = 0.011) phases, compared to the before-training phase. There was no significant difference in bias between the after-training and during-training phase (*t*_[23]_ = 1.89, *Bonferonni-corrected p* = 0.229).

### Variability with the trained auditory stimulus

Experimental phase also had a significant effect on variability (*F*_[2,44]_ = 17.06, *p* < 0.001). Feedback reliability did not have a significant effect (*F*_[1,22]_ = 0.26, *p* = 0.614), although there was a significant interaction between phase and feedback reliability (*F*_[2,44]_ = 5.65, *p* = 0.007): Participants trained with less reliable visual feedback showed significantly reduced variability during-training than before-training, whereas participants trained with more reliable visual feedback did not (see Supplementary Information S1). Figure 2B shows the mean variability for each of the experimental phases. Post hoc comparisons showed that variability was significantly reduced in training (*t*_[23] =_ 4.46, *Bonferonni-corrected p* < 0.001) and after-training (*t*_[23] =_ 4.17, *Bonferonni-corrected p* = 0.001) phases, compared to the pre-training phase. There was no significant change in variability between during-training and after-training phases (*t*_[23] =_ 0.74, *Bonferonni-corrected p* > 0.999).

### Bias and variability with the untrained auditory stimulus

Before- and after- training, all participants were also asked to localise the “more reliable” auditory stimulus (A1). Bias and variability of these judgments were analysed using repeated-measures ANOVAs with experimental phase (before- and after-training) as the within-subjects factor and visual feedback reliability (VF1, VF2) as the between-subject factor. Variability (*F*_[1,22]_ = 16.91, *p* < 0.001) but not bias (*F*_[1,22]_ = 3.89, *p* = 0.061) was significantly reduced in the after-training compared to before-training phase. There was no effect of visual feedback reliability on either bias (*F*_[1,22]_ = 0.04, *p* = 0.836) or variability (*F*_[1,22]_ = 0.09, *p* = 0.762), and no significant interactions (bias, *F*_[1,22]_ = 0.09, *p* = 0.769; variability, *F*_[1,22]_ = 0.54, *p* = 0.471). A1 bias and variability before- and after-training are plotted in Fig. 2.

We also asked whether the degree of change in bias or variability was different for the two auditory stimuli. Repeated-measures ANOVAs with experimental phase (before- and after-training) and stimulus (A1, A2) as the within-subjects factors, and visual feedback reliability (VF1, VF2) as the between-subject factor, indicated a significant interaction between experimental phase and stimulus for bias (*F*_[1,22]_ = 7.14, *p* = 0.014), but not variability (*F*_[1,22]_ = 0.63, *p* = 0.436). This reflected the finding reported above that bias was significantly reduced post-training for the “less reliable” (A2) stimulus, but not for the “more reliable” (A1) stimulus. There was no three-way interaction between experimental phase, stimulus and visual feedback, either in terms of bias (*F*_[1,22]_ = 0.87, *p* = 0.362) or variability (*F*_[1,22]_ = 0.75, *p* = 0.397). Note that measured A1 and A2 localisation biases were significantly greater than zero post-training (A1: *t*_[23]_ = 3.42, *p* = 0.002, A2: *t*_[23]_ = 5.26, *p* < 0.001), indicating that there was still scope for improvement.

**Figure 3:**
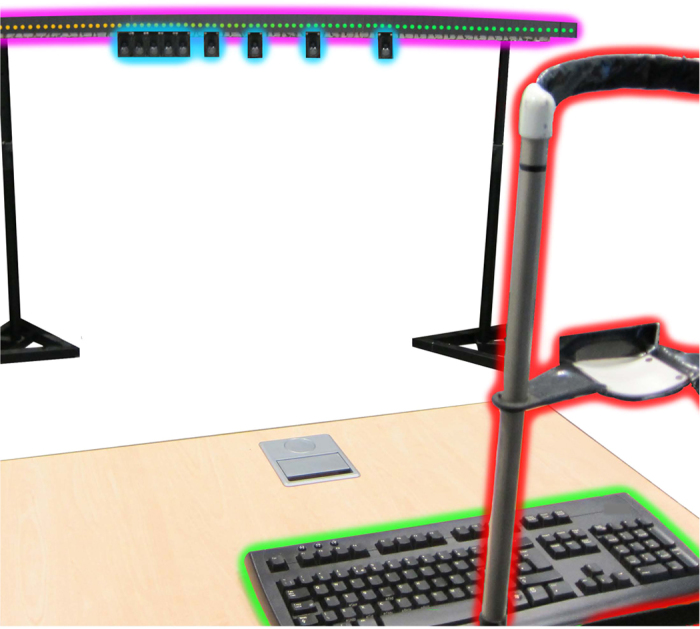
The Ring of LEDs and Speakers. During the experiment, the speakers (outlined in blue) were hidden using an acoustically transparent curtain to ensure that participants were unaware of their locations. Participants maintained their head position fixed at straight ahead, using a chin rest (outlined in red), and entered responses using a dial (not visible) and keyboard (outlined in green).

The difference in change in bias across A1/A2 stimuli indicates that the results were not simply explained by participants changing their response strategy. The shift for the A2 stimulus was greater, in line with this stimulus being more noisy (and being the one that was trained). The different changes in bias are not explained by a “floor effect” (both biases reaching near-zero from different starting points), as for both A1 and A2 post-learning biases were significantly greater than zero (A1: *t*_[23]_ = 3.42, *p* = 0.002, A2: *t*_[23]_ = 5.26, *p* < 0.001; see Fig. 3A).

## Discussion

This study aimed to assess (i) whether systematic biases in auditory localisation can be explained by reliance on a prior and (ii) whether auditory localisation biases can be reduced following training with accurate visual feedback. We found that when the variability (uncertainty) of auditory localisation estimates increased, biases also increased. This is consistent with observers relying on prior knowledge to reduce the uncertainty in their auditory location estimates. However, inconsistent with reliance on prior knowledge, we found that biases towards the periphery also increased with eccentricity, despite no corresponding increase in uncertainty. We also saw significant effects of training: Both biases and variability declined in during- and after- training phases compared to before- training.

Participants showed a tendency to overestimate the eccentricity of a sound source that could not be explained by their simply responding toward the mean of the stimulus set (as has been reported for other judgments^31^,^32^). This systematic overestimation of sound location eccentricity is consistent with many previously reported findings^1^,^13^,^14^,^33^, although underestimations have also been reported^3^. Previous studies have explained biases in terms of factors such as distorted spatial working memory, leading to biases for transient as opposed to on-going auditory targets^34^,^35^, or errors in accounting for the position of the eyes relative to the head, causing mismatches between eye-centred visual space and head-centred auditory space^13^. Differences in the persistence and magnitude of biases have also been documented across age groups^14^ and different auditory frequencies or bandwidths^1^,^7^. We found that participants made systematic transient sound localisation errors, even though we controlled and monitored eye position (as did Refs 1, 3). We also found that biases increased with increased auditory localisation uncertainty. This overall pattern is consistent with the BDT prediction that participants combining sensory information with prior knowledge will show greater biases under greater sensory uncertainty. However, the full pattern of results across locations was not well explained by a prior, because a prior biasing estimates to be more eccentric should become less strong (not more) as eccentricity increases. This suggests that biases in auditory localisation are both a function of uncertainty and some other, as yet unknown, parameter.

Interestingly, as with auditory localisation, we also found that reducing the reliability of visual localisation led to an overall overestimation in the eccentricity of the visual target. However, relative to auditory biases, visual biases were small (<2°). Previous studies have found evidence of the use of a common prior for specific visual and auditory judgments (although as noted there are problems with interpreting our own results solely in terms of priors). For example, humans systematically underestimate the speed of moving objects and moving sounds, in line with their reliance on a common prior that objects in the environment are more likely to be static^22^,^23^,^24^. Here, however, unlike for auditory localisation, overestimations of visual target eccentricity were not observed at all azimuths tested, and for the more reliable visual stimulus, there was actually a tendency to underestimate the eccentricity of targets presented at central positions. Previous studies have also reported biases in visual target localisation, and these have been shown to vary according to the position of eye gaze^11^, the presence of other visual targets^36^,^37^, spatial attention^38^ and retention interval^12^. When, as in the present study, participants had to maintain fixation and head position at straight ahead, both consistent (2–4°) overestimations across eccentricities^11^,^39^ and underestimations that increase (up to 4°) with eccentricity have been found^12^. There is a cortical magnification of central visual space, since the fovea is represented by a higher number of neurons than the periphery. It is possible that visual biases reflect combined effects of (i) sensory likelihoods biased towards central space because of incomplete accounting for this magnification and (ii) a prior biased towards the periphery. The influences of these may vary by task, stimulus, and location. We found a visual bias whose direction and magnitude differed at different eccentricities and for the different visual cues. The two visual stimuli chosen to manipulate visual localisation reliability also differed in the extent of the visual field that they covered: the “more reliable” stimulus had a mean width of 24 degrees, whereas the “less reliable” stimulus had a mean width of 13 degrees. Since factors including the spatial distance between elements on a display^37^ have been found to influence visual biases, it may be that differences in the width and spacing of visual stimuli may account for the differences in the visual bias we observed.

To perceive objects and events in the environment accurately, and to adapt to bodily and sensory changes during development and ageing, humans must keep sensory estimates calibrated. Errors or perceived mis-matches (e.g. in prism adaptation^15^) provide the feedback for this kind of learning. One question raised in the Introduction was why auditory localisation biases should be so prevalent, given lifelong opportunities to correct errors. A BDT explanation, which we investigated, proposes that such “errors” might reflect use of a prior. In the second part of our study we also conducted an initial test to assess whether the reliability of visual feedback during training influences any reduction in auditory localisation biases. Auditory localisation variability, for the trained (less reliable) and untrained (more reliable) auditory stimulus, was reduced in after-training compared to before-training phases, irrespective of the reliability of visual feedback. Reductions in localisation bias were also seen for both auditory stimuli, but the reduction was greater for the less reliable (trained) auditory stimulus. One possibility for this reduction in bias following training is that participants simply adjusted their response strategy, so that their localisation estimates were closer to the feedback that had been provided. However, had this been the case, we would have expected participants to have adjusted their responses similarly for both the more and less reliable auditory stimuli, which they did not. This could, however, also reflect a failure to generalise any learning with the trained (A2) stimulus to the untrained (A1) stimulus.

During training, participants could have attributed localisation errors signalled by visual feedback to systematic error (bias) – due to a mismatch in auditory and visual spatial representations – or random – due to visual or auditory sensory uncertainty. Consequently, we had expected that observers provided with more reliable visual feedback would be more likely to attribute errors to a mismatch in the auditory and visual mapping, as opposed to random sensory noise^29^, and would therefore show greater learning (faster improvements in accuracy). However, visual feedback reliability did not influence learning.

A recent study Odegaard, *et al*.^33^ used a different approach to examine whether biased sensory likelihoods and/or priors account for visual and auditory localisation biases. They asked participants to localise auditory (noise burst) and visual (Gaussian disk) stimuli presented separately or together at various azimuthal locations (−13, −6.5, 0, 6.5, or 13 degrees), and then determined which of six quantitative models, that varied in terms of sensory likelihood and/or prior parameters, best fitted their data. Results indicated that participants tended to underestimate the eccentricity of visual-only stimuli and, consistent with our findings, overestimate the eccentricity of auditory-stimuli. Auditory and visual biases under bimodal stimulus presentation were dependent on whether the observer inferred common or independent causes for the simultaneously presented auditory and visual stimuli. Unimodal (auditory-only, visual-only) and bimodal (auditory and visual) data were best accounted for by a model that incorporated eight parameters, including a centrally biased visual-only likelihood, a peripherally biased auditory-only likelihood and a general prior for centre. This model was superior to others that assumed non-biased sensory likelihoods and unimodal priors. In the present study, we used an experimental manipulation, instead of a modelling approach, to investigate whether a prior could account for biases in auditory localisation. We found that auditory-only biases increased as auditory-only localisation uncertainty increased, which is consistent with increased reliance on an auditory (or general) peripheral spatial prior. However, it is not clear how this prior could account for biases that increase with eccentricity, despite stable uncertainty. It may be that a model incorporating both biased sensory likelihoods and unimodal (visual-central, auditory-peripheral) priors may be necessary to fully account for auditory and visual localisation biases. Further research that combines behavioural data with modelling approaches is needed to address whether and why such priors and sensory representation biases exist and, as discussed above, investigate mechanisms other than Bayesian use of priors that might explain perceptual biases that increase with uncertainty.

In summary, previous research has found that humans show biases in auditory localisation of varying magnitude and direction^1^,^2^,^33^. Here, we found that participants showed a tendency to overestimate the eccentricity of a sound source, and that these overestimations increased as a function of localisation uncertainty. This is consistent with the Bayesian Decision Theory principle that as sensory uncertainty increased participants increasingly relied on prior information. However auditory localisation overestimations also increased with eccentricity and it is not clear how a simple prior favouring peripheral locations could account for this pattern. Additionally, we found that auditory localisation biases decreased across experimental phases, providing evidence that accuracy can be improved with experience (as well as precision). Further research is needed to evaluate which alternative mechanisms could explain increases of bias under sensory uncertainty.

## Methods

### Participants

24 adults aged 18 to 24 years (6 male, M = 20.5yrs, SD = 1.9yrs) with normal vision and normal hearing participated. Participants were recruited through the UCL psychology online subject pool. The study received approval from the London Hampstead research ethics committee and was conducted in accordance to the Declaration of Helsinki. Informed written consent was obtained from all participants prior to participation.

### Apparatus & Stimuli

Stimuli were presented using nine speakers (50 mm × 90 mm Visaton speakers SC 5.9) and up to 122 light-emitting diode pixels (Adafruit 12 mm diffused flat digital RGB LED pixels), mounted on a 2.5 m semi-circular ring (circle radius: 2.87 m), spanning −15 to +30 degrees (see Fig. 3). A further speaker was mounted on the wall, 20 degrees left of the ring. Stimulus presentation was controlled using Matlab (Version R2014a, The MathWorks Inc., Natick, Massachusetts, United States) and the Psychophysics toolbox extensions^40^,^41^,^42^, on a Windows 7 computer. The Matlab PsychPortAudio ASIO interface controlled audio presentation via a Focusrite Saffire PRO 40 (Focusrite plc, UK) sound card and audio signals were amplified using Lvpin Hi-Fi 2.1 (Lvpin Technology Co. Ltd, China) stereo amps. The sampling rate was 44.1 kHz and speakers were equalized for intensity using a sound level meter. An Arduino Uno microcontroller (SmartProjects, Strambino, Italy) was used to interface between the control computer and the LED pixels see ref. 43. Responses were made by rotating a dial (Griffin Technology PowerMate NA USB Controller) to control, (via Matlab), which LED pixel was illuminated. Eye position was monitored using a Tobii X120 (Tobii AB) eye tracker. An acoustically transparent curtain was arranged in front of the speakers.

Auditory stimuli were 100 msec (including 25 ms rise/fall time) band-pass-filtered noise bursts (tenth octave centred on 1000 Hz) presented at 50 dB SPL, (from speakers positioned at 0, 2, 6, 9, or 13 degrees, right of straight-ahead). These were hidden in background pink noise presented at 10 dB SPL (“more reliable” stimulus, which we denote *A1*) or 30 dB SPL (“less reliable”, stimulus, *A2*), (−35, 0, 1, 2, 3, 4, 6, 9, 13 and 18 degrees; mean position = 2**°;** mean position excluding speaker at −35**°** = 6**°**). Visual and visual feedback stimuli were 25 msec flashes of white light (4620 cd/m^2^) from either 45 (“more reliable”, *V1, VF1*) or 5 (“less reliable”, *V2, VF2*) LEDs, randomly sampled (on each trial, without replacement) based on a truncated normal distribution ranging from ±25 LEDs (with mean = 0, corresponding to the centre of the 50 LEDs, and standard deviation of 12 LEDs).

### Procedure

The experiment was divided into four tasks, split over two days (see Table 1). During each task, participants were asked to maintain their eye gaze at a fixation cue, consisting of two LEDs emitting red light (1300 cd/m^2^) presented at 0 degrees. A chin-rest was used to fix participants’ head position, and an eye-tracker was used to monitor eye position (a quick eye-tracking calibration task was completed before commencing the experiment). Participants initialized experimental tasks by pressing a keyboard key, and, provided that the eye-tracker detected that participants were fixating in the correct position, the trial would commence.

### Auditory and visual localisation before training

On the first day, participants completed an auditory localisation task and a visual localisation task. On each trial of the auditory localisation task, a brief noise burst was played at one of five speaker positions (0, 2, 6, 9, or 13 degrees relative to straight-ahead), while on each trial of the visual localisation task, a brief flash of light was presented from a sample of LEDs, the mean of which was centred at one of these same five positions (0, 2, 6, 9, 13, degrees). On each trial, following the stimulus presentation, two randomly selected adjacent LEDs (width spanning 1 degree) lit up. Participants were asked to move these two lights, by rotating the dial, toward the perceived source of the noise burst or light flash, maintaining their eyes fixed on the central fixation cue. Once participants were satisfied that the LEDs were aligned with the sound or flash location, they pressed a keyboard key to store their response and this immediately commenced the next trial. Each task consisted of four blocks of fifty trials (10 trials per location tested): two blocks with less reliable stimuli and two blocks with more reliable stimuli. Block order and stimulus location presentation was random. Prior to commencing the test blocks, participants completed a short practice block comprised of five trials (1 per location tested) with the more reliable stimulus.

### Training

On the second day, participants completed an auditory localisation task with visual feedback. As in the initial auditory localisation task, on each trial, a brief noise burst was played at one of the five speaker positions, following which participants adjusted the position of two white LEDs until they were aligned with the perceived sound source. However, on pressing a keyboard key to store their response, participants were presented a brief flash of light from a sample of LEDs whose mean position was centred at the veridical sound source location. Again, the task consisted of four blocks of fifty trials each. The auditory stimulus was the same across all blocks, corresponding to the “less reliable” (A2) stimulus in the previous task. Visual feedback stimuli were also the same across all blocks for each participant, but varied across participants (see Table 1): twelve participants were presented with a more reliable visual cue as feedback (VF1), and twelve were presented with a less reliable visual cue as feedback (VF2). The properties of these were the same as of V1 and V2 respectively during the visual localisation task.

### Auditory localisation after training

After training, the initial auditory localisation task was repeated exactly as before, with two auditory reliability levels (A1, A2) and without any feedback.

### Data Analysis

Trials during which the mean and/or standard deviation of eye coordinate position exceeded 2 degrees from the fixation target were excluded (<2% trials for any participant). Due to the size of the experimental set-up, participants’ localisation estimates were restricted to a maximum of 30 degrees. To account for this, truncated normal distributions (truncation point at 31 degrees) were fitted to each participant’s localisation estimates, at each location (0, 2, 6, 9, 13 degrees), for each stimulus (A1, A2, V1, V2). The mean and standard deviation of these distributions provided measures of each participant’s localisation bias (participant mean estimate – correct location) and variability, respectively. Biases at each position were then averaged across locations for a measure of mean bias. The standard deviation of deviances, at each position, were also averaged for a measure of mean variability (uncertainty).

## Additional Information

**How to cite this article**: Garcia, S. E. *et al*. Auditory Localisation Biases Increase with Sensory Uncertainty. *Sci. Rep.* **7**, 40567; doi: 10.1038/srep40567 (2017).

**Publisher's note:** Springer Nature remains neutral with regard to jurisdictional claims in published maps and institutional affiliations.

## Supplementary Material

Supplementary Information

## Figures and Tables

**Table 1:** Summary of the Experiment Phases, Tasks, and (Within-Subject & Between-Subject) Variables.

Day	Phase	Localisation Task	Within-Subject	Between-Subject
1	Before training	Auditory	Auditory reliability (A1, A2)	—
		Visual	Visual reliability (V1, V2)	—
2	Training	Auditory + visual feedback	None – all A2	Visual feedback (VF1, VF2)
	After training	Auditory	Auditory reliability (A1, A2)	(None, but analysed by VF)
