# Supplementary material for: Auditory Localisation Biases Increase with Sensory Uncertainty
Source: Sci Rep. 2017 Jan 11;7:40567. doi: 10.1038/srep40567 (PMC5225420; doi:10.1038/srep40567)
Supplement: Supplementary Information [file srep40567-s1.pdf]

Title

Auditory Localisation Biases Increase with Sensory Uncertainty

Authors

Sara E Garcia[1, 2, *], Pete R Jones[1, 2], Gary S Rubin[1, 2], Marko Nardini[3, 1, *]

(1) University College London, UCL Institute of Ophthalmology, London, UK

(2) NIHR Moorfields Biomedical Research Centre, London, UK

(3) Durham University, Department of Psychology, Durham, UK

* Corresponding authors

Email: sara.garcia.12@ucl.ac.uk, marko.nardini@durham.ac.uk

Supplementary Information

S1: Effect of visual feedback on bias and variability

Phase (before-, during, after- training) had a significant effect on bias ($F_{[1.38,30.26]} = 12.44$, $p < 0.001$), but visual feedback reliability did not ($F_{[1,22]} = 0.02$, $p = 0.896$), and there was no interaction between visual feedback and phase ($F_{[1.38,30.26]} = 0.97$, $p = 0.360$; see Fig. S1A). Phase also had a significant effect on variability ($F_{[2,44]} = 17.06$, $p < 0.001$), but again visual feedback reliability did not ($F_{[1,22]} = 0.26$, $p = 0.614$). However, there was a significant interaction between phase and visual feedback reliability on localisation variability ($F_{[2,44]} = 5.65$, $p = 0.007$): Participants trained with less reliable visual feedback, showed significantly reduced variability during-training than before-training ($t_{[11]} = 6.19$, $p < 0.001$), while for participants trained with more reliable visual feedback, the reduction in variability was not significant ($t_{[11]} = 2.00$, $p = 0.071$).

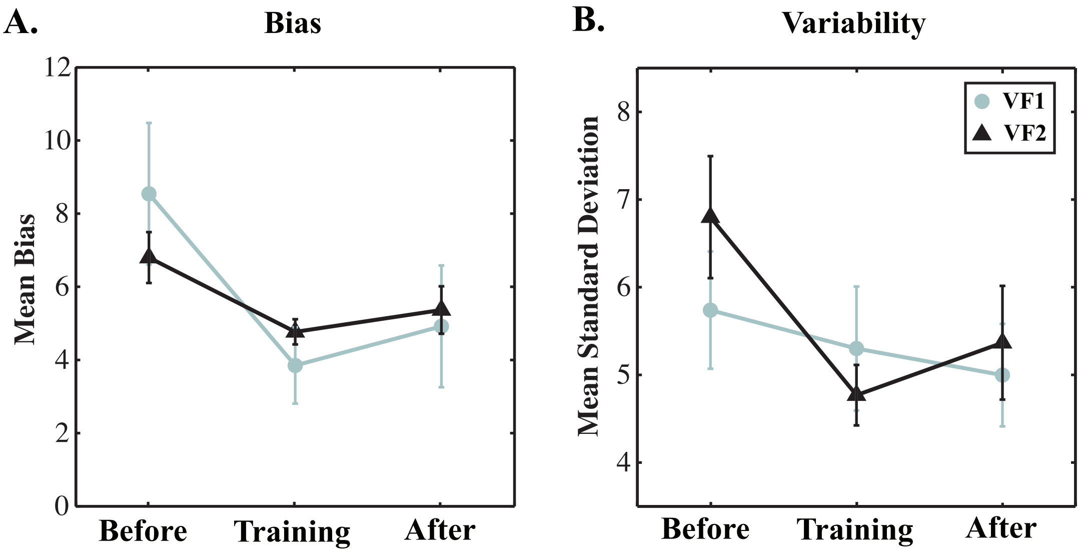

Figure S1: Mean Bias (A) and Mean Variability (B) Before-, During- and After- Training for Participants trained with More Reliable Visual Feedback (VF1) and Less Reliable Visual Feedback (VF2). Bars represent standard error of the mean.

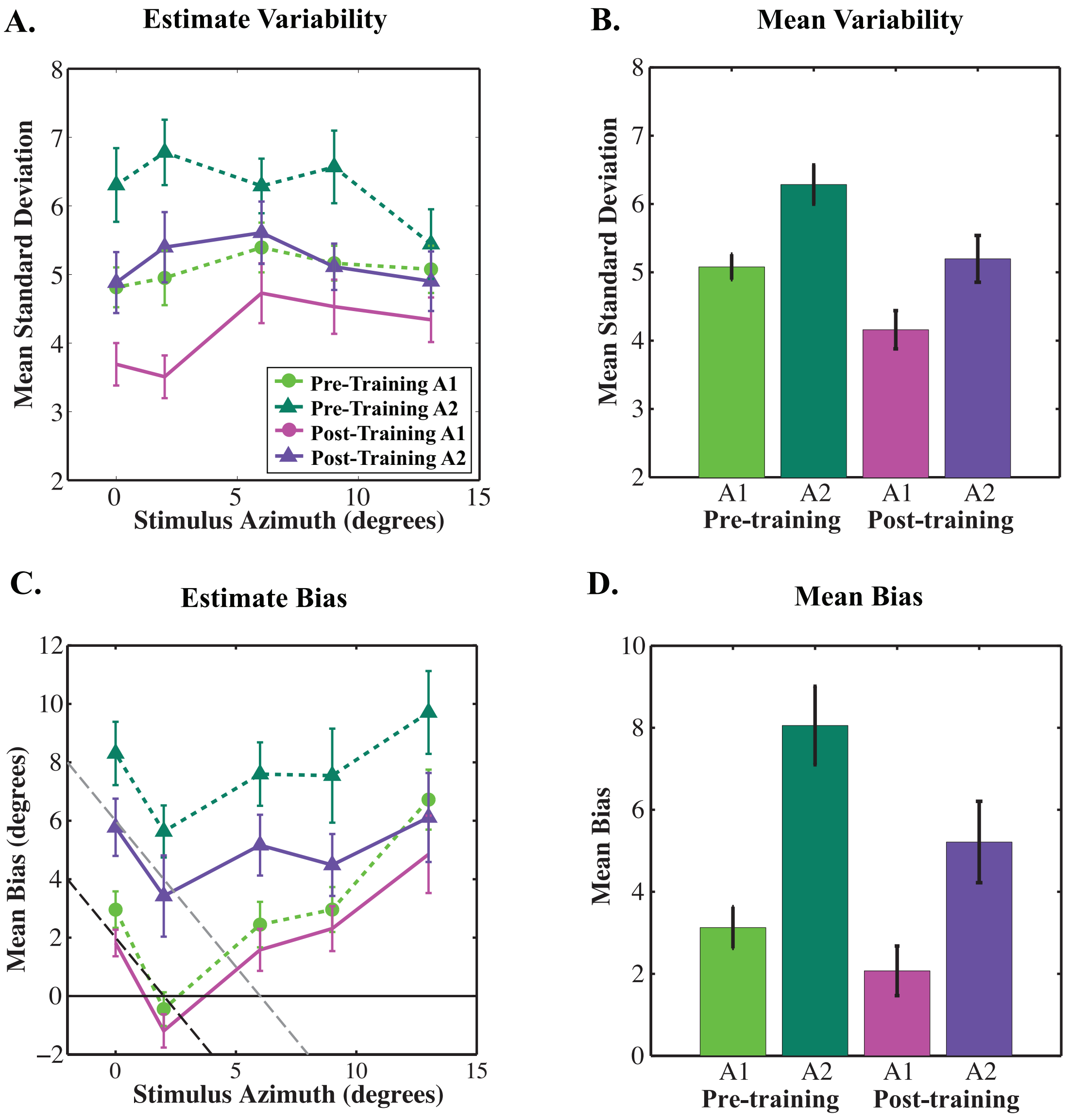

Figure S2: Mean Bias and Variability for Localizing Auditory and Visual Stimuli Before- and After-Training. A. Mean variability at each location for each stimulus. B. Mean variability at each location for each stimulus. C. Mean bias at each location for each stimulus. D. Mean bias for each stimulus across all locations tested. Grey dotted line in C indicates the line predicted by responding according to the mean of the target stimulus set. Black dotted line in C indicates the line predicted by responding according to the mean of the speakers presenting background noise. Error bars represent the standard error of the mean.
